# Tumor necrosis factor-α-induced protein 8-like 2 mRNA in peripheral blood mononuclear cells is associated with the disease progression of chronic hepatitis B virus infection

**DOI:** 10.1186/s12985-019-1224-7

**Published:** 2019-10-28

**Authors:** Yi Liu, Jia Jin, Jian Ji, Xi-Mei Gao, Yu-Chen Fan

**Affiliations:** 1 Department of Gastroenterology, Shanxian Central Hospital, Heze, 274399 China; 2 Department of Cardiology, Zhangqiu District People’s Hospital of Jinan, Jinan, 250200 China; 3 Department of Clinical Laboratory, Qilu Hospital of Shandong University, Jinan, 250012 China; 4 Department of International Medicine, Qilu Hospital of Shandong University, Wenhuaxi Road 107#, Jinan, 250012 China; 5 Department of Hepatology, Qilu Hospital of Shandong University, Wenhuaxi Road 107#, Jinan, 250012 China; 6 Department of Immunology, Shandong University School of Basic Medical Science, Shandong University, Wenhuaxi Road 44#, Jinan, 250012 Shandong China

**Keywords:** Tumor necrosis factor-α-induced protein 8-like 2, Chronic hepatitis B, Liver cirrhosis, Hepatocellular carcinoma, Receiver operating characteristic

## Abstract

**Background:**

Tumor necrosis factor-alpha-induced protein 8-like 2 (TIPE2) is a novel target and molecule in the negative regulation of immune homeostasis. The present study aimed to investigate the dynamic expression of TIPE2 mRNA during the progression of chronic hepatitis B virus (HBV) infection.

**Methods:**

A total of 193 patients with chronic HBV infection were retrospectively recruited into this cross-sectional study, including 97 patients with chronic hepatitis B (CHB), 55 with liver cirrhosis and 41 with HBV-related hepatocellular carcinoma (HCC). TIPE2 mRNA was determined using real-time quantitative polymerase chain reaction.

**Results:**

The expression of TIPE2 levels in patients with HCC was significantly decreased compared with expression in patients with liver cirrhosis, CHB and healthy controls (*P* < 0.05); meanwhile, the TIPE2 mRNA levels in patients with CHB and liver cirrhosis were significantly increased compared with levels in healthy controls (*P* < 0.01). In liver cirrhosis, the TIPE2 mRNA level in the decompensated state was significantly higher than that in the compensated state (*P* < 0.05). In HCC patients, TIPE2 mRNA was significantly associated with venous invasion, tumor size and tumor node metastasis stage. Furthermore, the optimal cutoff of 0.78 for the level of TIPE2 mRNA has a sensitivity of 97.56% and a specificity of 88.16% for discriminating HCC from patients with CHB and liver cirrhosis.

**Conclusions:**

TIPE2 mRNA was associated with various stages of chronic HBV infection, ranging from CHB to liver cirrhosis and HCC. Furthermore, TIPE2 mRNA with an optional cutoff value of 0.78 might serve as a promising biomarker to discriminate HBV-associated HCC from CHB and LC patients.

## Background

Hepatitis B virus (HBV) infection is a global health problem, and approximately 2 billion people are infected with HBV, of whom more than 350 million are chronically infected [1]. The natural course of chronic HBV infection often ranges from chronic hepatitis B (CHB) to liver cirrhosis (LC) and eventually to hepatocellular carcinoma (HCC) [2]. Persistent HBV infection and liver cirrhosis have been identified as leading risk factors for the development of HCC [3–5]. It is well demonstrated that the interaction of host and HBV, especially the strength of the immune response post HBV infection, contributes to the progression of HBV infection [6].

TIPE2, tumor necrosis factor-alpha-induced protein 8 (TNFAIP8)-like 2 (TNFAIP8L2), is a newly identified negative regulator of innate immunity and cellular immunity [7]. TIPE2 can maintain immune homeostasis via negative regulation of signaling by binding to T cell receptors (TCRs) and Toll-like receptors (TLRs), and knockout of the tipe2 gene in mice leads to multiorgan inflammation, splenomegaly and premature death [7]. The high-resolution crystal structure reveals that TIPE2 contains a centrally located, large, hydrophobic central cavity, which appears to be a death effector domain (DED)-like structure, and the topology differs from caspase-8 [8]. Similar to other TNFAIP8 proteins, TIPE2 interacts with caspase-8 through their respective DED domains and promotes factor-associated suicide (Fas)-induced apoptosis [7]. In addition, TIPE2 has also been reported to control innate immunity to bacteria and double-stranded RNA (dsRNA) viruses [9], and downregulation of TIPE2 is associated with increased phagocytosis and bacterial killing [10]. Furthermore, it has been shown that TIPE2 binds to the rat sarcoma (Ras)-interacting domain to inhibit Ras-induced tumorigenesis [11]. These results suggest that TIPE2 may not only be involved in inflammation but also in cancer development.

Accelerating evidence supports the hypothesis that aberrant TIPE2 expression might play an important role in the development and progression of chronic inflammatory diseases, autoimmune disorders, stroke, diabetic nephropathy, carcinoma and atherosclerosis [12–21]. Recent studies have revealed the downregulation of TIPE2 expression in HCC tissue compared with the paired adjacent nontumorous tissue [21]. However, the expression profiling of TIPE2 mRNA in peripheral blood mononuclear cells (PBMCs) of patients at different stages of chronic HBV infection has not yet been reported. Therefore, we aimed to investigate the dynamic expression of TIPE2 mRNA during the progression of chronic HBV infection in the present study.

## Materials and methods

### Patients and samples

The study consisted of 41 patients with HCC, 52 patients with LC, 97 patients with CHB and 14 healthy controls (HCs) from the Department of Hepatology and Department of International Medicine, Qilu Hospital of Shandong University, between December 2013 and May 2015. CHB patients were defined as persistent elevation of alanine aminotransaminase (ALT) levels and positive hepatitis B surface antigen (HBsAg) for at least 6 months prior to the beginning of this study [22]. The diagnosis of LC was based on abdominal images and clinically relevant portal hypertension or hepatic encephalopathy. Liver cirrhosis has been subdivided into compensated LC and decompensated LC. The compensated LC subgroup was based on the definition of CHB and the following: (1) computed tomography or ultrasonography images combined with laboratory findings or (2) fibrosis score 4 in liver histopathology. Decompensated LC was defined as the appearance of ascites, variceal bleeding, hepatic encephalopathy, and/or jaundice on the basis of compensated LC [23]. HCC patients were diagnosed according to the 2010 update of the American Association for the Study of Liver Diseases (AASLD) Practice Guidelines for Management of hepatocellular carcinoma [24]. Exclusion criteria included the following: (1) coinfection with human immunodeficiency virus, hepatitis C virus, hepatitis D virus and autoimmune or metabolic liver disease; (2) treatment-free interval of less than 1 year; (3) hematologic disorders; (4) severe alcohol abuse; and (5) coexisting with other tumors. All participants gave written informed consent under protocols approved by the local Research and Ethics Committee at Qilu Hospital of Shandong University, in accordance with the guidelines of the 1975 Declaration of Helsinki [25].

### RNA and complementary DNA (cDNA) preparation from PBMCs

Five milliliters of peripheral blood from all subjects was collected into EDTA anticoagulated tubes. PBMCs were separated using Ficoll-Paque Plus (GE Healthcare, Uppsala, Sweden) density gradient centrifugation. Total RNA from PBMCs was extracted using TRIzol reagent (Invitrogen, Carlsbad, CA, USA) according to the manufacturer’s instructions. cDNA was synthesized from two micrograms of RNA using a first-strand cDNA synthesis kit (Fermentas, Vilnius, Lithuania).

### Quantitative real-time PCR

Primers for TIPE2 were forward 5′-GGAACATCCAAGGCAAGACTG-3′ and reverse 5′-AGCACCTCACTGCTTGTCTCATC-3′. Primers for β-actin were forward 5′- ATGGGTCAGAAGGATTCCTATGTG-3′ and reverse 5′- CTTCATGAGGTAGTCAGTCAGGTC-3′. β-Actin was used as the endogenous control. Real-time PCR was performed using a SYBR Premix Ex TaqTM (Takara, Toyobo, Japan) according to the manufacturer’s instructions. The PCR reaction was performed according to the following thermal profile: denaturation at 95 °C for 30 s, followed by 40 cycles of 95 °C for 5 s, 60 °C for 30 s and 72 °C for 30 s. Each sample was carried out in triplicate. Data analysis was performed with the LightCycler 480 Software (Roche Diagnostics, Roche Applied Science, Mannheim, Germany), and the results were determined using the comparative (2^-△△Ct^) method.

### Clinical pathological data collection

The serum biochemical markers (COBAS integra 800, Roche Diagnostics, Germany) included aspartate aminotransferase (AST), alanine aminotransferase (ALT), total bilirubin (TBIL), albumin (ALB), and creatinine (Cr). Hemostasis markers (ACL TOP 700, Instrument Laboratory, USA) included the prothrombin activity (PTA) and international normalized ratio (INR). Hematological markers (Sysmex XE-2100, Sysmex Corporation, Kobe, Japan) included white blood cell (WBC), hemoglobin (HGB) and platelet (PLT) counts. The level of α-fetoprotein (AFP) was measured by an automatic analyzer (COBAS e 601, Roche Diagnostics, Roche Applied Science, Mannheim, Germany). Hepatitis B virus serologies, including HBsAg, hepatitis B e antigen (HBeAg), and anti-HBe were measured by an automatic analyzer (cobas 6000 analyzer series, Roche Diagnostics, Rotkreuz, Switzerland). These markers were measured using standard laboratory methods in the Department of Clinical Laboratory, Qilu Hospital, Shandong University. The serum viral load of HBV DNA was quantified using a PCR System (ABI 7300, Applied Biosystems, Foster City, CA, USA) with a detection sensitivity of 500 IU/ml.

The Model for End-Stage Liver Disease (MELD) scores were calculated according to the Malinchoc formula [26]: MELD score = 9.57 × loge (creatinine [mg/dL]) + 3.78 × loge (bilirubin [mg/dL]) + 11.2 × loge (INR) + 6.43 × (etiology: 0 if cholestatic or alcoholic, 1 otherwise).

### Statistical analysis

The Kolmogorov-Smirnov test was performed to determine whether the data fit a normally distributed population. Quantitative variables were described as medians (25th percentile; 75th percentile). Categorical variables were expressed as numbers (percentages). The Kruskal-Wallis test or Mann–Whitney *U*-test was used to compare the nonparametric quantitative variables within groups. The chi-square test was used to compare the categorical data. The correlation between variables was evaluated using the Spearman rank correlation test. All tests were two-tailed, and a *P* < 0.05 was considered statistically significant. All statistical analyses were performed using IBM SPSS 19.0 software (SPSS Inc., Chicago, IL, USA).

## Results

### General characteristics

**Fig. 1:**
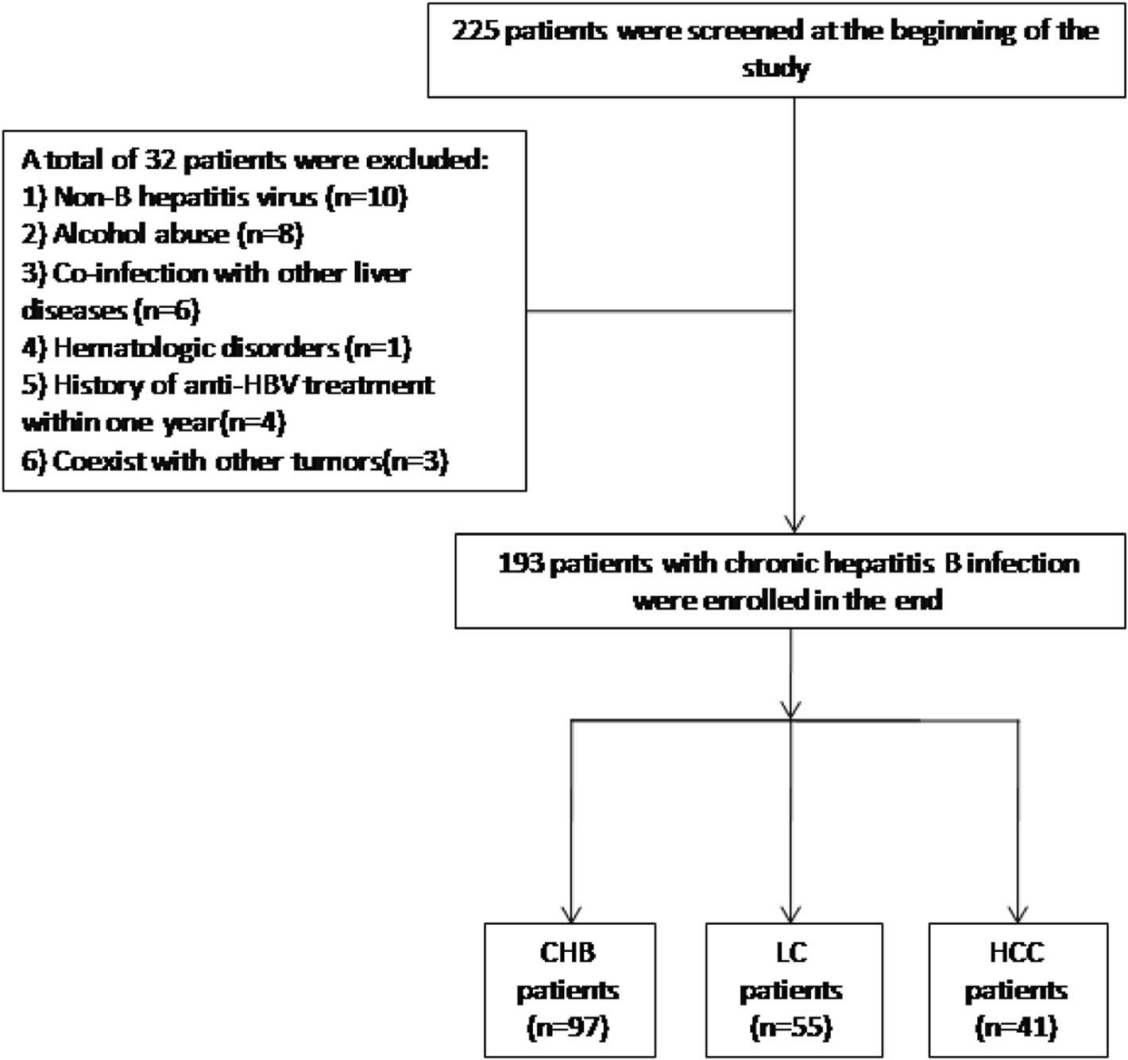
The flowchart of enrolled patients

The flowchart for the inclusion and exclusion of all patients is shown in Fig. 1. A total of 193 patients were included in three groups: 97 patients with CHB, 55 patients with LC, and 41 patients with HCC. The baseline characteristics of the enrolled patients and healthy controls are shown in Table 1.

**Table 1:** Baseline characteristics of the enrolled patients

Variable	HCC group (*n* = 41)	LC group (*n* = 55)	CHB group (*n* = 97)	HC group (*n* = 14)
Sex (male/female)	35/6	40/15	72/25	10/4
Age (years)	60 (52.7–64.2)	51 (41–59)	41 (29.5–48.5)	45 (32.7–49.7)
HBeAg(−/+)	24/17	28/27	34/63	NA
HBVDNA(−/+)	19/22	17/38	19/78	NA
HBsAg	4879 (2490–6796)	4781 (2515–6819)	3769 (1264–5728.5)	NA
ALT (U/L)	51 (32.7–99.3)	42 (26–89)	118 (43–272.5)	17.5 (12–25.2)
AST (U/L)	86 (49–178.5)	57 (39–85)	56 (33–134)	13.5 (12–17.2)
TBIL (umol/L)	24.9 (14.8–50.9)	30.4 (18.7–60.2)	18.5 (11.6–46.25)	11 (8.7–13.2)
ALB (g/L)	35.8 (32.8–39.9)	32.7 (29.1–38.1)	41.3 (37.4–44.5)	45 (44.5–54)
PTA (%)	75 (66–80.3)	68 (55–80)	94 (86–103)	84.5 (79.5–90.5
AFP (ng/ml)	444.5 (254.5–1693.5)	25.8 (58.9–181.27)	181.2 (8.2–118.3)	12 (96.6–13)
WBC (E+ 09/L)	5.5 (3.9–9.6)	5.8 (3.3–6.7)	5.3 (4.6–6.4)	5.8 (5.3–7.0)
HGB (g/L)	129 (110–143.5)	129 (101–140)	150 (140.5–155)	141.5 (137.9–146)
PLT (E+ 09/L)	134 (98.7–211.3)	98 (78–154)	188 (154.3–229.5)	193 (172.5–206)

Quantitative variables are expressed as the median (25th percentile; 75th percentile)

*CHB* Chronic hepatitis B, *HCs* Healthy controls, *HBsAg* Hepatitis B surface antigen, *HBeAg* Hepatitis B e antigen, *ALT* Alanine aminotransferase, *AST* Aspartate aminotransferase, *ALB* Albumin, *TBIL* Total bilirubin, *PTA* Prothrombin activity, *AFP* α-fetoprotein, *WBC* White blood cell, *HGB* Hemoglobin, *PLT* Platelet, *NA* Not available

### Expression profile of TIPE2 mRNA from PBMCs of HBV-infected patients

**Fig. 2:**
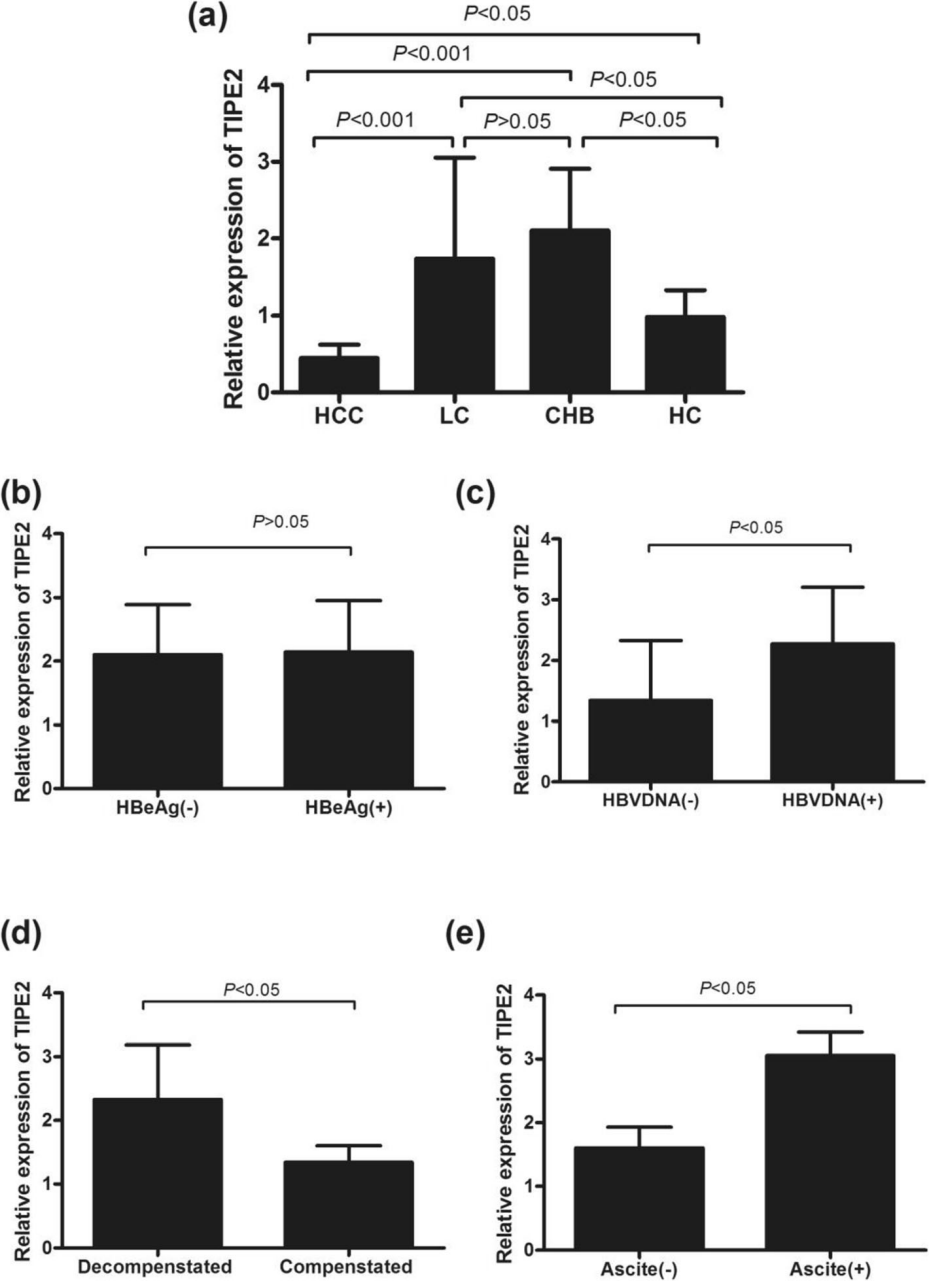
Relative expression of TIPE2 mRNA from peripheral blood mononuclear cells in different stages of chronic HBV infection and healthy controls. **a** TIPE2 expression from PBMCs of CHB, LC, and HBV-associated HCC patients and healthy controls; **b**-**c** expression of TIPE2 mRNA in CHB patients with and without HBeAg and HBVDNA positive; **d** expression of TIPE2 mRNA in compensated and decompensated LC patients; **e** expression of TIPE2 mRNA in LC patients with and without ascites

The levels of TIPE2 mRNA were significantly upregulated in patients with CHB and LC compared with the levels in healthy controls (CHB: 2.1[1.24, 2.91] vs. 1.00[0.94, 1.33], *P* < 0.01; LC: 1.74[1.34, 3.05] vs. 1.00[0.94, 1.33], *P* < 0.01), whereas there were no significant differences between CHB patients and LC patients. TIPE2 mRNA was significantly downregulated in HCC patients compared with the levels in LC (0.45[0.30–0.61] vs. 1.74[1.34, 3.05], *P* < 0.05), CHB (0.45[0.30–0.61] vs. 2.1[1.24, 2.91], *P* < 0.05), and healthy controls (0.45[0.30–0.61] vs. 1.00[0.94, 1.33], *P* < 0.05) (Fig. 2a). Furthermore, Figs. 2b-c) show that there were no significant differences in TIPE2 mRNA levels between HBeAg-positive patients and HBeAg-negative patients (2.10 [1.21, 2.88] vs. 2.14 [1.25, 2.95], *P* > 0.05). However, the level of TIPE2 mRNA in HBVDNA-positive patients was significantly higher compared with that in HBVDNA-negative patients (2.27 [1.38, 3.20] vs. 1.34 [0.84, 2.33], *P* < 0.05).

**Fig. 3:**
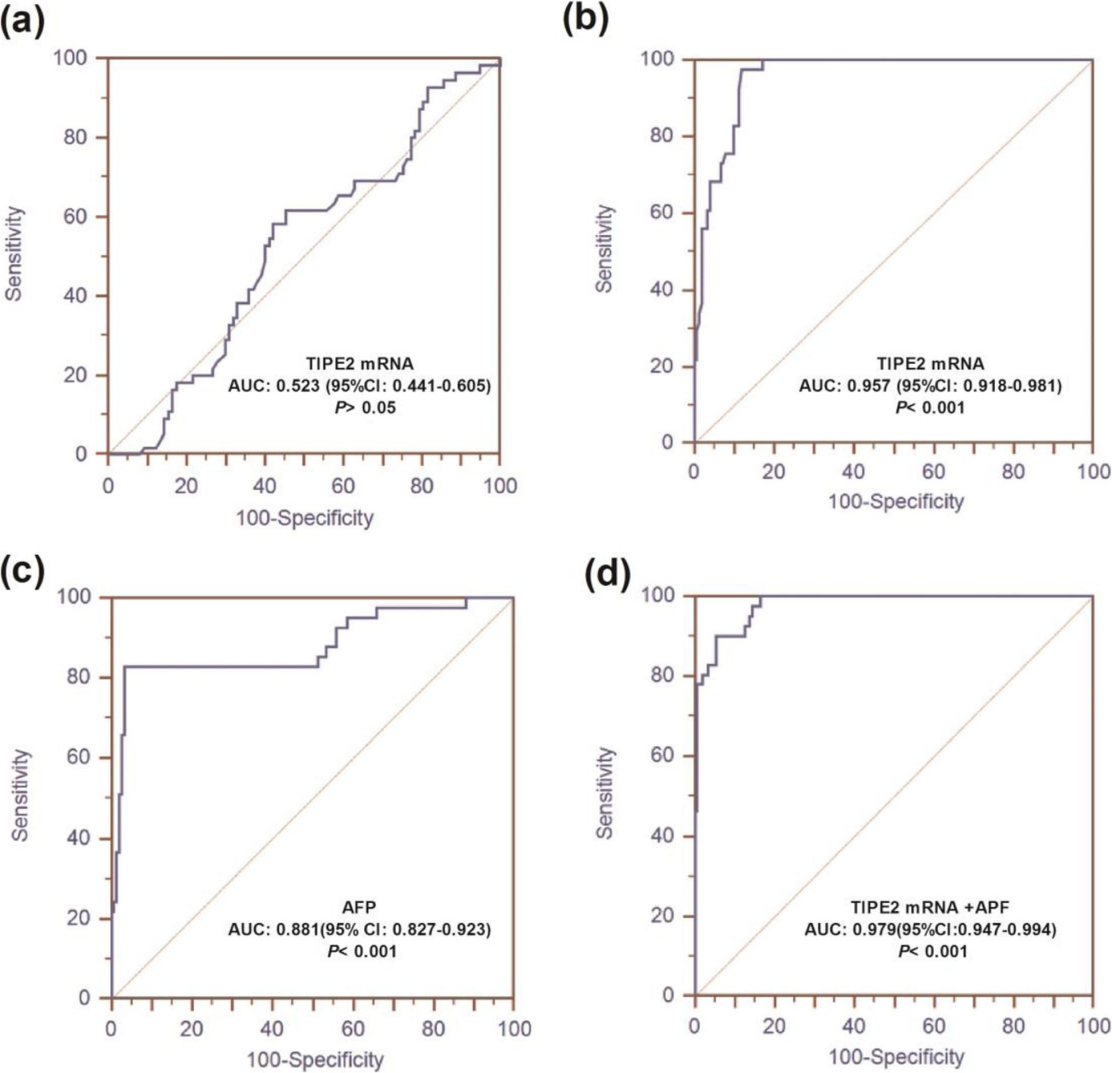
ROC curve analysis of TIPE2 mRNA. **a** TIPE2 mRNA for differentiating CHB patients from LC patients; **b** TIPE2 mRNA for differentiating HBV-associated HCC from CHB and LC patients; **c** AFP alone for differentiating HBV-associated HCC from CHB and LC patients; **d** AFP in combination with TIPE2 mRNA for differentiating HBV-associated HCC from CHB and LC patients

In LC patients, Fig. 2d shows that the TIPE2 mRNA level in decompensated LC patients was higher than that in compensated LC patients (2.33[1.44, 3.18] vs. 1.36 [0.92, 1.74], *P* < 0.05). Furthermore, there was a significant difference in TIPE2 mRNA between LC patients with ascites and without ascites (3.05[1.43, 3.42] vs. 1.63 [1.21, 1.91], *P* < 0.05), as shown in Fig. 3e.

In HCC patients, the expression of TIPE2 mRNA had a significant relationship with venous invasion, tumor size and tumor node metastasis (TNM) stage. Lower TIPE2 mRNA expression tends to show a higher incidence of venous invasion or larger tumor size. In addition, TIPE2 mRNA was downregulated in HCC patients with advanced TNM stage (III-IV) compared with the mRNA in those with early TNM stage (I-II). However, TIPE2 mRNA was not significantly related to age, sex, HBeAg, liver cirrhosis, multiple primary tumor number and serum AFP level (Table 2).

**Table 2:** Relationship between TIPE2 expression in PBMCs and clinical pathological parameters of HCC patients

Parameter	Total number	Expression of TIPE2	*P* value
Sex			
Male	35	0.45 [0.23,0.56]	0.08
Female	6	0.59 [0.46,0.75]	
Age (years)			
≤ 60	23	0.45 [0.31,0.67]	0.713
>60	18	0.45 [0.22,0.53]	
HBeAg			
Negative	24	0.43 [0.3,0.61]	0.968
Positive	17	0.45 [0.25,0.62]	
Cirrhosis			
Absent	20	0.45 [0.30,0.52]	0.327
Present	21	0.45 [0.27, 0.72]	
AFP ng/ml			
≤ 400	23	0.48 [0.34,0.67]	0.134
>400	18	0.38 [0.22,0.56]	
Venous invasion			
Negative	19	0.52 [0.45, 0.75]	0.002
Positive	22	0.35 [0.19,0.52]	
Primary tumor number			
Single	29	0.45 [0.23, 0.64]	0.703
Multiple	12	0.42 [0.32,0.54]	
Tumor size			
≤ 5 cm	18	0.56 [0.35,0.75]	0.007
>5 cm	23	0.39 [0.23,0.47]	
TNM staging			
I/II	27	0.54 [0.45,0.76]	0.001
III/IV	14	0.35 [0.23,0.52]	

Quantitative variables are expressed as the median (25th percentile; 75th percentile)

*HBeAg* Hepatitis B e antigen, *AFP* α-fetoprotein, *TNM* Tumor node metastasis

### The diagnostic value of TIPE2 mRNA in various stages of chronic HBV infection

Figure 3 shows that the areas under the receiver operating characteristic (ROC) curve analysis (AUROC) of TIPE2 mRNA for the discrimination of CHB and LC was 0.523 (95% confidence interval [CI] 0.441–0.605, *P* > 0.05) with a sensitivity of 61.82%, a specificity of 54.64%, a positive predictive value (PPV) of 43.6% and a negative predictive value (NPV) of 71.6%, indicating that TIPE2 mRNA might not be an optimal marker for the discrimination between CHB and LC. Furthermore, TIPE2 mRNA had an AUC of 0.957 (95% CI: 0.918–0.981, *P* < 0.001), and the optimal cutoff was 0.78 with a sensitivity of 97.56%, a specificity of 88.16%, a PPV of 69% and an NPV of 99.3% in discriminating HBV-associated HCC from CHB and LC patients. In addition, the serum AFP level yielded an AUROC of 0.881 (95% CI: 0.827–0.923, *P* < 0.001) with a sensitivity of 82.93%, a specificity of 96.71%, a PPV of 87.2% and an NPV of 95.5% in the diagnosis of HCC from CHB and LC. The AUROC of TIPE2 mRNA combined with AFP was 0.979 (95% CI: 0.947–0.994, *P* < 0.001), with a sensitivity of 90.24%, a specificity of 94.74%, a PPV of 82.2% and an NPV of 97.3%.

### Correlations between TIPE2 mRNA and clinical parameters in chronic HBV infection

**Fig. 4:**
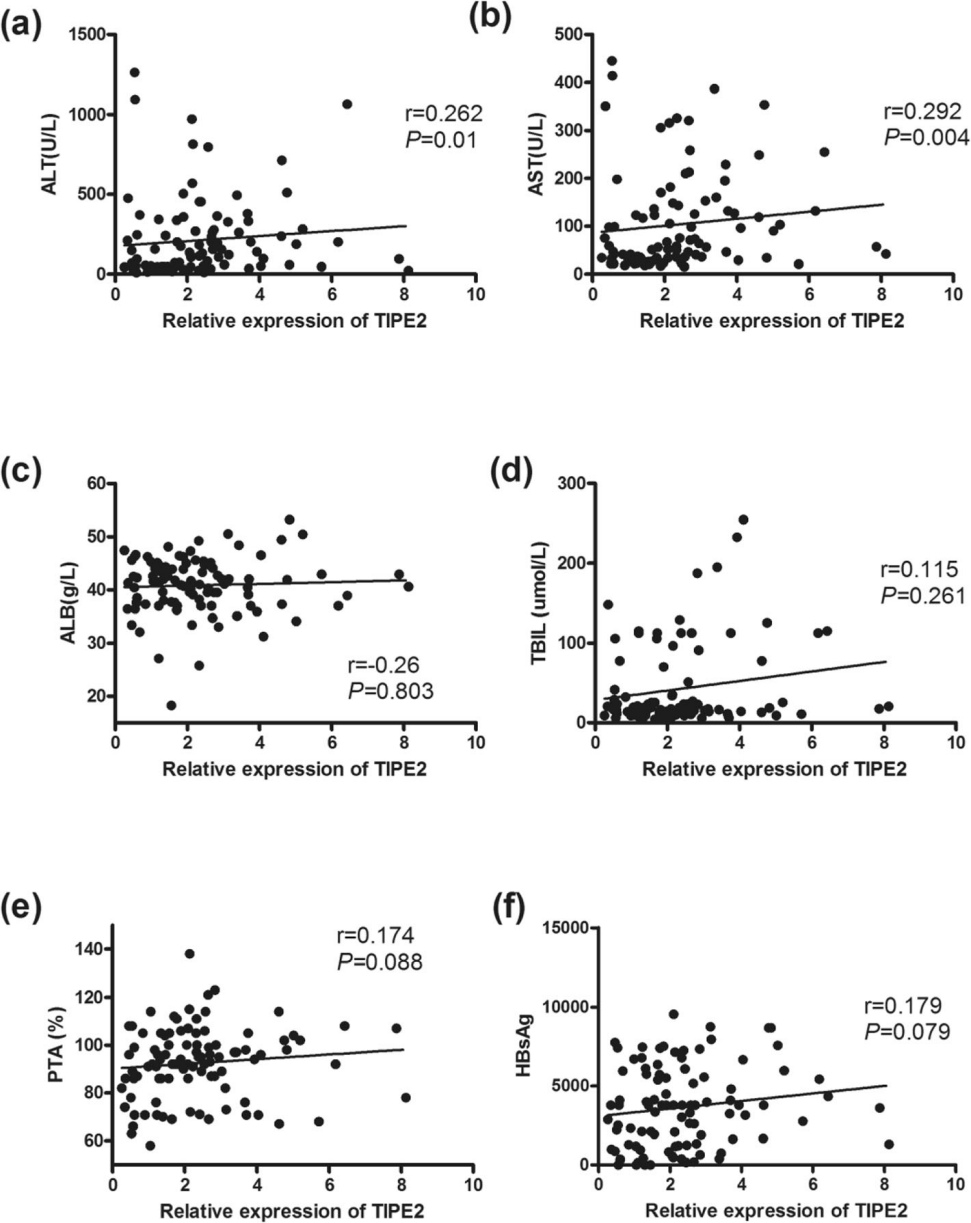
Expression of TIPE2 mRNA from peripheral blood mononuclear cells in CHB patients. **a** Correlation between the TIPE2 mRNA level and ALT level (*r* = 0.262, *P* < 0.05); **b** correlation between the TIPE2 mRNA level and AST level (*r* = 0.292, *P* < 0.05); **c** correlation between the TIPE2 mRNA level and ALB level (*r* = − 0.26, *P* = 0.803); **d** correlation between the TIPE2 mRNA level and TBIL (*r* = 0.115, *P* = 0.261); **e** correlation between the TIPE2 mRNA level and PTA (*r* = 0.174, *P* = 0.088); **f** correlation between the TIPE2 mRNA level and HBsAg (*r* = 0.179, *P* = 0.079)

**Fig. 5:**
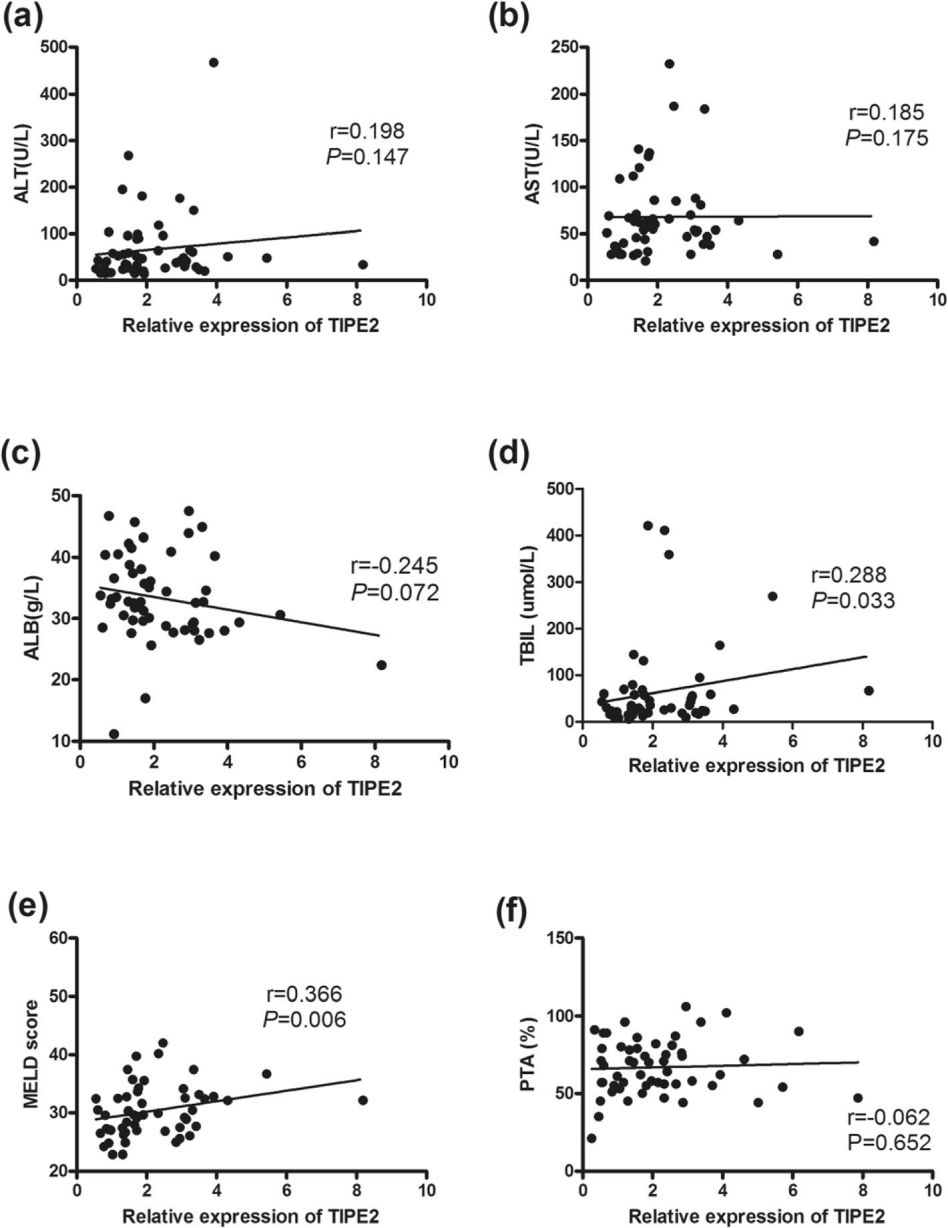
Expression of TIPE2 mRNA from peripheral blood mononuclear cells in LC patients. **a** Correlation between the TIPE2 mRNA level and ALT level (*r* = 0.198, *P* = 0.147); **b** correlation between the TIPE2 mRNA level and AST level (*r* = 0.185, *P* = 0.175); **c** correlation between the TIPE2 mRNA level and ALB level (*r* = − 0.245, *P* = 0.072); **d** correlation between the TIPE2 mRNA level and TBIL (*r* = 0.288, *P* = 0.033); **e** correlation between the TIPE2 mRNA level and MELD score (*r* = 0.366, *P* = 0.006); **f** correlation between the TIPE2 mRNA level and PTA (*r* = − 0.062, *P* = 0.652)

**Fig. 6:**
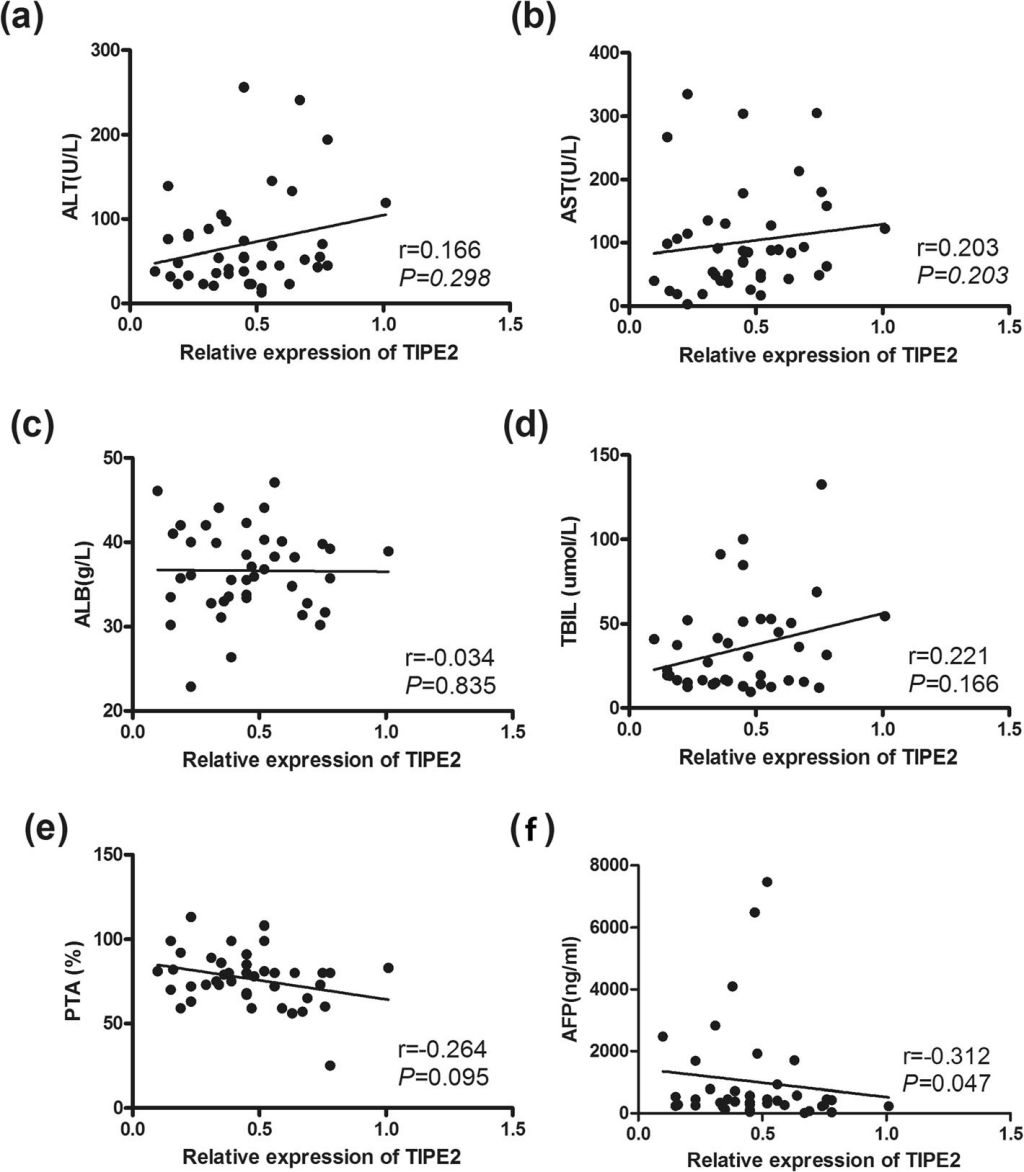
Expression of TIPE2 mRNA from peripheral blood mononuclear cells of HBV-associated HCC patients. **a** Correlation between the TIPE2 mRNA level and ALT level (*r* = 0.166, *P* = 0.298); **b** correlation between the TIPE2 mRNA level and AST level (*r* = 0.203, *P* = 0.203); **c** correlation between the TIPE2 mRNA level and ALB level (*r* = − 0.034, *P* = 0.835); **d** correlation between the TIPE2 mRNA level and TBIL (*r* = 0.221, *P* = 0.166); **e** correlation between the TIPE2 mRNA level and PTA (*r* = − 0.264, *P* = 0.095); **f** correlation between the TIPE2 mRNA level and AFP (*r* = − 0.312, *P* = 0.047)

We further analyzed the correlations between TIPE2 mRNA and clinical parameters, including serum ALT, ALT, ALB, TBIL, PTA, MELD score, AFP and HBsAg. TIPE2 mRNA was positively correlated with serum ALT and AST (*r* = 0.262, *P* < 0.05; *r* = 0.292, *P* < 0.05) (Fig. 4). In addition, TIPE2 mRNA was found to be significantly positively correlated with TBIL and the MELD score in LC patients (*r* = 0.288, *P* = 0.033; *r* = 0.366, *P* < 0.01) (Fig. 5) and negatively correlated with serum AFP levels in HCC patients (*r* = 0.312, *P* < 0.05) (Fig. 6).

## Discussion

Chronic HBV infections can cause immune-mediated liver damage progressing to cirrhosis and hepatocellular carcinoma (HCC) [27]. Chronic persistent inflammation in the liver may play a critical role in carcinogenesis [28–30]. TIPE2 is a newly identified negative regulator in maintaining immune homeostasis. However, the potential roles of TIPE2 mRNA at different stages of HBV-associated liver diseases have not yet been reported. In this study, we determined the dynamic expression of TIPE2 mRNA in PBMCs in different stages of HBV-associated liver diseases and identified the potential diagnostic value of TIPE2 mRNA as a biomarker for discriminating HBV-associated HCC from CHB and LC patients.

We first reported the dynamic expression of TIPE2 mRNA during the natural history of chronic HBV infection, ranging from CHB and LC to HCC. In the present study, we demonstrated that the median levels of TIPE2 mRNA in chronic hepatitis B were significantly higher compared with those in healthy controls. These results might disagree with the previous report by Xi W et al. in 2011 in which the authors reported that TIPE2 mRNA of CHB patients was significantly downregulated compared with that of normal controls [12]. In their study, the authors defined chronic hepatitis B as HBsAg for more than 6 months but not considered liver inflammation and the immune response. Therefore, our previous study in 2017 aimed to determine the mRNA and protein levels of TIPE2 in peripheral blood and liver from CHB patients [31]. Interestingly, we reported that TIPE2 in immune clearance phases was higher than that in the immune tolerance phase, whereas TIPE2 in HBeAg-negative hepatitis was higher than that in the low replication phase, indicating that TIPE2 might contribute to the immune clearance of CHB patients [31]. In the present study, CHB patients were selected as HBsAg for more than 6 months and had elevated ALT levels, indicating that CHB patients are normally in the immune stages of immune clearance and have HBeAg-negative hepatitis. Therefore, this issue might be the reason why we obtained a discrepancy in TIPE2 expression in different populations of CHB patients [12, 31]. In addition, the healthy controls in the present study were relatively small (*n* = 14 for our study; *n* = 21 for Xi’s study [12]) for comparison, although the baseline characteristics of CHB patients and healthy controls were well matched with respect to age and sex. A larger and multicenter study with well-matched controls will be helpful in the future.

Liver cirrhosis is defined as the histological development of regenerative nodules surrounded by fibrous bands in response to chronic liver injury, which leads to portal hypertension and end-stage liver disease [32]. This is the first report to investigate the expression of TIPE2 in HBV-associated liver cirrhosis. We demonstrated that the relative level of TIPE2 mRNA in liver cirrhosis was similar to that in CHB patients, whereas the TIPE2 mRNA level in decompensated LC patients was higher than that in compensated LC patients. Furthermore, there was a significant difference in TIPE2 mRNA between LC patients with ascites and without ascites. These results indicated that TIPE2 might contribute to the progression of LC patients from the compensated phase into the decompensated phase. The MELD score is the current widely popular tool for predicting short-term mortality of liver cirrhosis [33]. We reported that TIPE2 mRNA is positively correlated with the MELD score in liver cirrhosis. This result also supports the hypothesis that TIPE2 might play an important role in the severity of liver cirrhosis. In other nonviral liver cirrhosis, TIPE2 might also exert its unique role. For example, decreased expression of TIPE2 has been reported to be associated with the hyperreactivity of monocytes to toll-like receptor ligands in primary biliary cirrhosis [34]. Functional analysis showed that TIPE2 might have protective effects on liver fibrosis by reversing the activated hepatic stellate cells [35]. Therefore, these results strongly suggest that TIPE2 mRNA is associated with liver cirrhosis. However, the exact mechanism of TIPE2 in liver cirrhosis should be well investigated in future studies.

HCC is one of the most common and rapidly fatal human malignancies in the world [36]. In the present study, we reported that the downregulation of TIPE2 mRNA expression was significantly associated with venous invasion, primary tumor size and TNM stage. In recent years, increasing studies have demonstrated that TIPE2 plays an inhibitory role in human cancer development [6, 37, 38]. A recent study showed that TIPE2 can suppress the growth and aggressiveness of hepatocellular carcinoma cells through downregulation of the phosphoinositide 3-kinase/AKT signaling pathway [39]. Another report showed that TIPE2 is an endogenous inhibitor of Rac1 in HCC, which results in attenuation of invasion and metastasis of HCC [21]. In addition, Erk1/2 and NF-κB activation has also been demonstrated to be involved in HCC progression [40]. These data suggest that TIPE2 might serve as a new target for HCC therapy.

In addition, ROC analysis has been performed to evaluate whether TIPE2 mRNA could serve as a noninvasive diagnostic biomarker. Our data showed that TIPE2 mRNA is superior to AFP for diagnosing HBV-associated HCC from CHB and LC patients, while the combination of TIPE2 mRNA and AFP conferred no advantage over TIPE2 mRNA alone for detecting HBV-associated HCC from CHB and LC patients. These results suggested that TIPE2 mRNA could serve as a useful noninvasive biomarker to diagnose HBV-associated HCC from CHB and LC patients. However, there are no data on the diagnosis of TIPE2 in the discrimination of HCC from LC and CHB patients in another cohort. Therefore, the diagnostic value of TIPE2 should be validated in more cohorts from other countries and other races in future studies. In fact, our present study is a type of cross-sectional study with the results showing that TIPE2 expression is higher in CHB and CHB with cirrhosis but lower in CHB with HCC. It seems reasonable to speculate that CHB patients with lower TIPE2 expression might be at high risk for the incidence of HCC. However, a further prospective consecutive cohort of CHB patients in a large population is essential for identifying the epidemiological effects of TIPE2 on the occurrence of HCC.

## Conclusions

In summary, TIPE2 mRNA was associated with the disease progression of chronic HBV infection. TIPE2 mRNA might serve as a potential noninvasive biomarker to discriminate HBV-associated HCC from CHB and LC patients. However, the diagnostic value of TIPE2 should be validated and investigated in more cohorts from other countries and other races in future studies.

## Data Availability

The data analyzed during the current study are available from the corresponding author on reasonable request.

## Abbreviations

AFP: α-fetoprotein
ALB: Albumin
ALT: Alanine aminotransferase
AST: Aspartate aminotransferase
AUROC: Areas under the ROC curves
CHB: Chronic hepatitis B
CI: Confidence interval
HBsAg: Hepatitis B surface antigen
HBV: Hepatitis B virus
HCC: Hepatocellular carcinoma
HGB: Hemoglobin
LC: Liver cirrhosis
MELD: Model for End-Stage Liver Disease
NPV: Negative predictive value
PBMCs: Peripheral blood mononuclear cells
PLT: Platelet
PPV: Positive predictive value
PTA: Prothrombin activity
PT-INR: Prothrombin-international normalized ratio
ROC: Receiver operating characteristic
TBIL: Total bilirubin
TCRs: T cell receptors
TIPE2: Tumor necrosis factor-α-induced protein 8-like 2
TLRs: Toll-like receptors
WBC: White blood cell

## References

[1] Scaglione SJ, Lok ASF (2012). Effectiveness of Hepatitis B treatment in clinical practice. Gastroenterology.

[2] Arzumanyan A, Reis HM, Feitelson MA (2013). Pathogenic mechanisms in HBV- and HCV-associated hepatocellular carcinoma. Nat Rev Cancer.

[3] Neuveut C, Wei Y, Buendia MA (2010). Mechanisms of HBV-related hepatocarcinogenesis. J Hepatol.

[4] Chemin I, Zoulim F (2009). Hepatitis B virus induced hepatocellular carcinoma. Cancer Lett.

[5] Perz JF, Armstrong GL, Farrington LA, Hutin YJF, Bell BP (2006). The contributions of hepatitis B virus and hepatitis C virus infections to cirrhosis and primary liver cancer worldwide. J Hepatol.

[6] Ji J, Zhang YY, Fan YC (2019). TIPE2 as a potential therapeutic target in chronic viral hepatitis. Expert Opin Ther Targets.

[7] Sun H, Gong S, Carmody RJ, Hilliard A, Li L, Sun J, Kong L, Xu L, Hilliard B, Hu S (2008). TIPE2, a negative regulator of innate and adaptive immunity that maintains immune homeostasis. Cell.

[8] Zhang X, Wang J, Fan C, Li H, Sun H, Gong S, Chen YH, Shi Y (2009). Crystal structure of TIPE2 provides insights into immune homeostasis. Nat Struct Mol Biol.

[9] Sun H, Zhuang G, Chai L, Wang Z, Johnson D, Ma Y, Chen YH (2012). TIPE2 controls innate immunity to RNA by targeting the phosphatidylinositol 3-kinase-Rac pathway. J Immunol.

[10] Wang Z, Fayngerts S, Wang P, Sun H, Johnson DS, Ruan Q, Guo W, Chen YH (2012). TIPE2 protein serves as a negative regulator of phagocytosis and oxidative burst during infection. Proc Natl Acad Sci U S A.

[11] Gus-Brautbar Y, Johnson D, Zhang L, Sun H, Wang P, Zhang S, Zhang L, Chen YH (2012). The anti-inflammatory TIPE2 is an inhibitor of the oncogenic Ras. Mol Cell.

[12] Xi WJ, Hu YJ, Liu YG, Zhang J, Wang L, Lou YW, Qu ZH, Cui J, Zhang GZ, Liang XH (2011). Roles of TIPE2 in hepatitis B virus-induced hepatic inflammation in humans and mice. Mol Immunol.

[13] Kong L, Liu K, Zhang YZ, Jin M, Wu BR, Wang WZ, Li W, Nan YM, Chen YH (2013). Downregulation of TIPE2 mRNA expression in peripheral blood mononuclear cells from patients with chronic hepatitis C. Hepatol Int.

[14] Li D, Song LJ, Fan YC, Li X, Li YJ, Chen J, Zhu FL, Guo C, Shi YY, Zhang LN (2009). Down-regulation of TIPE2 mRNA expression in peripheral blood mononuclear cells from patients with systemic lupus erythematosus. Clin Immunol.

[15] Lou YW, Sun HH, Morrissey S, Porturas T, Liu SX, Hua XX, Chen YHH (2014). Critical roles of TIPE2 protein in murine experimental colitis. J Immunol.

[16] Zhang Y, Wei XB, Liu LX, Liu SX, Wang ZY, Zhang B, Fan BX, Yang F, Huang SY, Jiang F (2012). TIPE2, a novel regulator of immunity, protects against experimental stroke. J Biol Chem.

[17] Zhang SY, Zhang Y, Wei XB, Zhen JH, Wang ZY, Li MY, Miao W, Ding H, Du PC, Zhang WC (2010). Expression and regulation of a novel identified TNFAIP8 family is associated with diabetic nephropathy. Biochim Biophys Acta Mol basis Dis.

[18] Zhao Q, Zhao M, Dong T, Zhou C, Peng Y, Zhou X, Fan B, Ma W, Han M, Liu S (2015). Tumor necrosis factor-alpha-induced Protein-8 Like-2 (TIPE2) Upregulates p27 to decrease Gastic Cancer cell proliferation. J Cell Biochem.

[19] Zhang G, Zhao L, Wang Y, Shao J, Cui J, Lou Y, Geng M, Zhang N, Chen YH, Liu S (2015). TIPE2 protein prevents injury-induced restenosis in mice. Biochim Biophys Acta.

[20] Lou Y, Liu S, Zhang C, Zhang G, Li J, Ni M, An G, Dong M, Liu X, Zhu F (2013). Enhanced atherosclerosis in TIPE2-deficient mice is associated with increased macrophage responses to oxidized low-density lipoprotein. J Immunol.

[21] Cao X, Zhang L, Shi Y, Sun Y, Dai S, Guo C, Zhu F, Wang Q, Wang J, Wang X (2013). Human tumor necrosis factor (TNF)-alpha-induced protein 8-like 2 suppresses hepatocellular carcinoma metastasis through inhibiting Rac1. Mol Cancer.

[22] Lok AS, McMahon BJ (2009). Chronic hepatitis B: update 2009. Hepatology.

[23] Thabut D, Bureau C, Layese R, Bourcier V, Hammouche M, Cagnot C, Marcellin P, Guyader D, Pol S, Larrey D (2019). Validation of Baveno VI criteria for screening and surveillance of esophageal Varices in patients with compensated cirrhosis and a sustained response to antiviral therapy. Gastroenterology.

[24] Bruix J, Sherman M (2011). Management of hepatocellular carcinoma: an update. Hepatology.

[25] Hellmann F, Verdi M, Schlemper BR, Caponi S (2014). 50th anniversary of the declaration of Helsinki: the double standard was introduced. Arch Med Res.

[26] Williams Felicity R., Berzigotti Annalisa, Lord Janet M., Lai Jennifer C., Armstrong Matthew J. (2019). Review article: impact of exercise on physical frailty in patients with chronic liver disease. Alimentary Pharmacology & Therapeutics.

[27] Seeger C, Mason WS (2015). Molecular biology of hepatitis B virus infection. Virology.

[28] Park O, Wang H, Weng H, Feigenbaum L, Li H, Yin S, Ki SH, Yoo SH, Dooley S, Wang F-S (2011). In vivo consequences of liver-specific Interleukin-22 expression in mice: implications for human liver disease progression. Hepatology.

[29] Shlomai A, de Jong YP, Rice CM (2014). Virus associated malignancies: the role of viral hepatitis in hepatocellular carcinoma. Semin Cancer Biol.

[30] Ji J, Eggert T, Budhu A, Forgues M, Takai A, Dang H, Ye Q, Lee J-S, Kim JH, Greten TF, Wang XW (2015). Hepatic stellate cell and monocyte interaction contributes to poor prognosis in hepatocellular carcinoma. Hepatology.

[31] Fan YC, Zhang YY, Wang N, Sun YY, Wang K (2017). Tumor necrosis factor-alpha-induced protein 8-like 2 (TIPE2) is associated with immune phases of patients with chronic hepatitis B. Oncotarget.

[32] Knolle PA, Thimme R (2014). Hepatic immune regulation and its involvement in viral hepatitis infection. Gastroenterology.

[33] Salerno F, Merli M, Cazzaniga M, Valeriano V, Rossi P, Lovaria A, Meregaglia D, Nicolini A, Lubatti L, Riggio O (2002). MELD score is better than child-Pugh score in predicting 3-month survival of patients undergoing transjugular intrahepatic portosystemic shunt. J Hepatol.

[34] Qin B, Wei T, Wang L, Ma N, Tang Q, Liang Y, Yang Z, Zhou L, Zhong R (2016). Decreased expression of TIPE2 contributes to the hyperreactivity of monocyte to toll-like receptor ligands in primary biliary cirrhosis. J Gastroenterol Hepatol.

[35] Xu DD, Li XF, Li YH, Liu YH, Huang C, Meng XM, Li J (2018). TIPE2 attenuates liver fibrosis by reversing the activated hepatic stellate cells. Biochem Biophys Res Commun.

[36] Sherman M (2010). Hepatocellular Carcinoma: Epidemiology, Surveillance, and Diagnosis. Semin Liver Dis.

[37] Padmavathi G, Banik K, Monisha J, Bordoloi D, Shabnam B, Arfuso F, Sethi G, Fan L, Kunnumakkara AB (2018). Novel tumor necrosis factor-alpha induced protein eight (TNFAIP8/TIPE) family: functions and downstream targets involved in cancer progression. Cancer Lett.

[38] Niture S, Dong X, Arthur E, Chimeh U, Niture SS, Zheng W, Kumar D (2018). Oncogenic role of tumor necrosis factor alpha-induced protein 8 (TNFAIP8). Cells.

[39] Wang L, Chen C, Feng S, Tian J (2018). TIPE2 suppresses growth and aggressiveness of hepatocellular carcinoma cells through downregulation of the phosphoinositide 3kinase/AKT signaling pathway. Mol Med Rep.

[40] Zhang YH, Yan HQ, Wang F, Wang YY, Jiang YN, Wang YN, Gao FG (2015). TIPE2 inhibits TNF-alpha-induced hepatocellular carcinoma cell metastasis via Erk1/2 downregulation and NF-kappaB activation. Int J Oncol.

